# Effectiveness of Peripheral Nerve Block for Early Active Exercise: Three Cases of Pyogenic Flexor Tenosynovitis

**DOI:** 10.1155/cro/4789432

**Published:** 2025-06-30

**Authors:** Takahiro Sato, Tsuyoshi Shirahata, Koji Nozaka, Naohisa Miyakoshi

**Affiliations:** Department of Orthopedic Surgery, Akita University, Akita, Japan

## Abstract

To prevent limitations in the range of motion after hand surgery, it is crucial to minimize scar tissue formation and adhesions. Thus, it is important to initiate active hand exercises during the early postoperative period. Peripheral nerve blocks (PNBs) may be employed for active hand exercises as they minimize postoperative pain. We reported three cases of pyogenic flexor tendonitis in which PNB was administered postoperatively, and changes in the range of motion were observed. In all cases, a pulp–palmar distance (PPD) of 1 mm was achieved within 1 week after surgery under PNB, and all cases were evaluated as “good” in Flynn's functional evaluation at the last observation. However, PPD worsened at 3 weeks postoperatively, and all patients exhibited limited proximal interphalangeal joint extension at the final observation. Although our method effectively improved the flexion range of motion, additional measures should be taken to improve the extension range of motion.

## 1. Introduction

Hand surgeries can limit the range of finger motion due to scar tissue formation and adhesions. Active exercise immediately after surgery is essential [1]. Selective peripheral median and ulnar nerve blocks (NBs) are useful for relieving postoperative pain and promoting early active exercise [1–3]. These peripheral nerve blocks (PNBs) are administered in the distal one-third of the forearm to relieve pain, allowing for active hand exercises that preserve the movement of the extrinsic muscles. PNB has been used postoperatively in various hand surgeries (i.e., phalangeal fractures, pyogenic arthritis of the digits, and pyogenic flexor tenosynovitis (PFT)). PFT is an infection of the synovial sheath surrounding the flexor tendon of the digit, typically developing after a penetrating wound through hematogenous dissemination or direct extension of infection from neighboring tissues. Initial treatment strategies include early administration of broad-spectrum intravenous antibiotics and urgent surgical intervention [4]. Despite appropriate treatment, PFT can lead to joint contractures (10%–25%) [5]. Therefore, management, including postoperative rehabilitation, is important to minimize range of motion (ROM) limitations. We hypothesized that early active exercise under PNB could effectively prevent postoperative contractures due to PFT. To date, only a few case reports on PNB [2, 3] have been reported. Additionally, only the visual analog scale and ROM at the last observation were included in the endpoints, and the progress of ROM was not revealed. We report three cases in which changes in the postoperative ROM in patients who underwent PNB were monitored using the pulp–palmar distance (PPD).

## 2. Case Presentation

**Figure 1:**
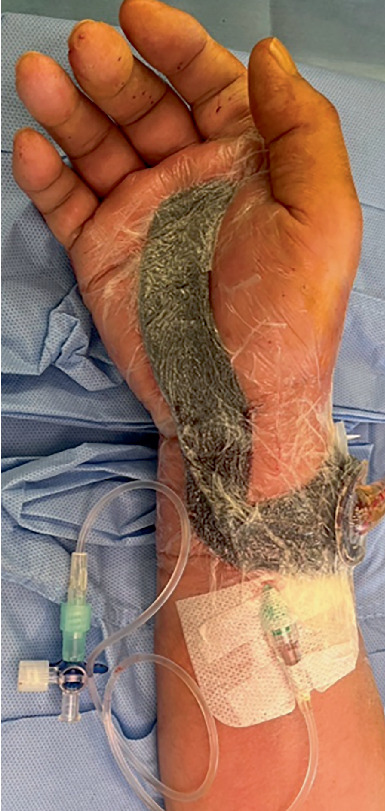
Appearance of wrist block (catheter implantation).

We report three cases (one man and two women, ages 16–81 years) of patients who underwent PNB (Table 1). To assess the severity of PFT, we used Michon's classification [6] as follows: Stage 1, increased clear fluid in the sheath and synovial hyperemia; Stage 2, turbid or purulent fluid and synovial granuloma; and Stage 3, necrosis of the tendon, sheath, or pulleys. PNB was conducted as follows: we administered 5 mL of 0.25% levobupivacaine to either the median nerve, ulnar nerve, or both nerves at the level of the wrist. We selected the nerves to be anesthetized according to the affected fingers. The method of administration involved direct injection using a needle or catheter (Figure 1). A catheter implantation was performed immediately after surgery. The injection was given once a day in the morning as a single shot. The administration of anesthesia started 1 day after surgery, and the duration of administration was adjusted according to the patient's ROM and condition. In all patients, active hand exercises were allowed a day after surgery. We instructed the patients to perform hand exercises consisting of active flexion and extension 10 times every 2 h as self-rehabilitation. In addition to self-rehabilitation, all patients underwent postoperative rehabilitation with a hand therapist: 5 days a week, 20 min once a day during hospitalization, and 20 min once a week as an outpatient. No restrictions were placed on either active or passive movement.

**Figure 2:**
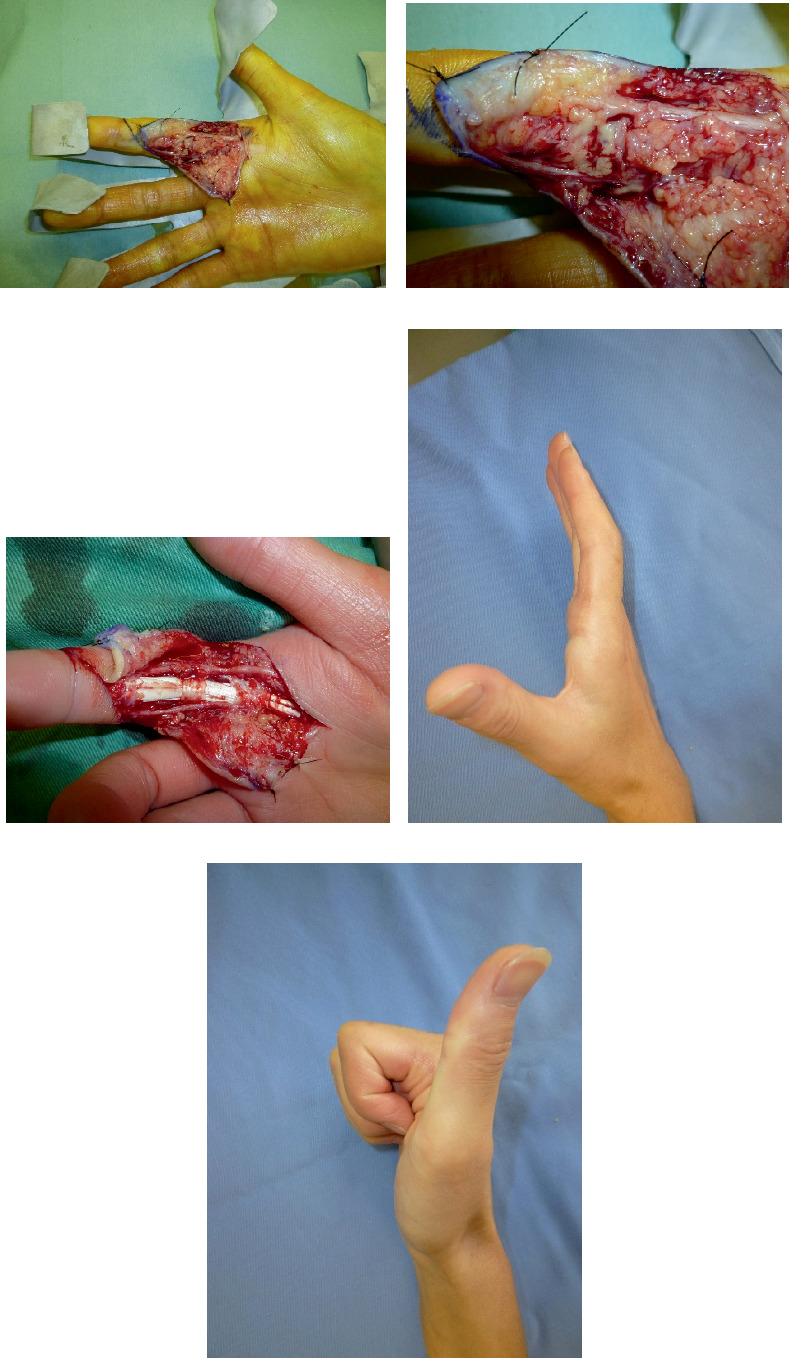
Photographs of Case 1. (a, b) Before debridement. (c) After debridement. (d, e) Appearance at last observation.

Case 1 was of a 16-year-old girl whose index finger was bitten by a cat. On the day of the injury, she was examined by a physician and treated with medications, but swelling and pain gradually increased. The patient was referred to our department for further treatment 4 days after the injury. Kanavel's four cardinal signs (tenderness throughout the sheath, flexion posture of the finger, exquisite pain on extending the finger, and uniform swelling of the involved finger) were observed. She was diagnosed with PFT and underwent surgery. A skin incision was created from the middle of the index finger to the palmar cutaneous line, which was expanded to the flexor tendon. Synovial hyperplasia and pus were observed around one bite wound (Figure 2a,b). The tendons did not melt. Michon's classification determined as Stage 2. Cleaning, synovectomy, and debridement were then performed (Figure 2c). Postoperatively, antibiotics were administered intravenously and orally for 2 weeks. Culture results showed *Pasteurella multocida*. PNB of the median nerve was performed for 10 days. The patient's postoperative course was uneventful. At the last observation, proximal interphalangeal (PIP) joint extension was −10°, but PPD was 0 cm (Figure 2d,e).

Case 2 was of an 81-year-old woman whose middle finger was injured by a chestnut thorn. Swelling and pain gradually worsened after the injury, and she visited a doctor 9 days later. The following day, she was referred to our department for further treatment. Kanavel's four cardinal signs were observed, leading to a diagnosis of PFT. Cleaning and synovectomy were performed on the same day. The puncture wound was distal to the interphalangeal joint, and pus was observed around the flexor tendon at depth. The tendons remained intact. Michon's classification determined as Stage 2. Culture results showed *Streptococcus pyogenes*. Postoperatively, antibiotics were administered intravenously and orally for 16 days, and PNB of the median nerve was performed for 10 days. The patient's postoperative course was uneventful. At the last observation, PIP joint extension was −30°, but PPD was 0 cm.

Case 3 was of a 49-year-old man whose little finger was cut with a kitchen knife. Swelling and pain gradually worsened after the injury, and the patient visited a doctor 5 days later. Two days later, he was referred to our department for further treatment due to nonimprovement. Kanavel's four cardinal signs were noted during the physical examination. Cleaning and synovectomies were performed on the same day. An incision was created along the PIP cutaneous line of the little finger, from the distal interphalangeal cutaneous line to the carpal, and extended to the flexor tendon. There was infected synovium and turbid fluid but no pus or melting of the tendon. Michon's classification determined as Stage 2. The culture results were negative. We diagnosed PFT based on the cause of injury, physical examination, and intraoperative findings. Postoperatively, antibiotics were administered intravenously and orally for 7 days. PNB of the ulnar nerve was performed for 5 days. The patient's postoperative course was uneventful. The ROM at the last observation was metacarpophalangeal (MP) joint extension +10, PIP joint extension −45, distal interphalangeal joint extension 0, and PPD 0 cm.

**Figure 3:**
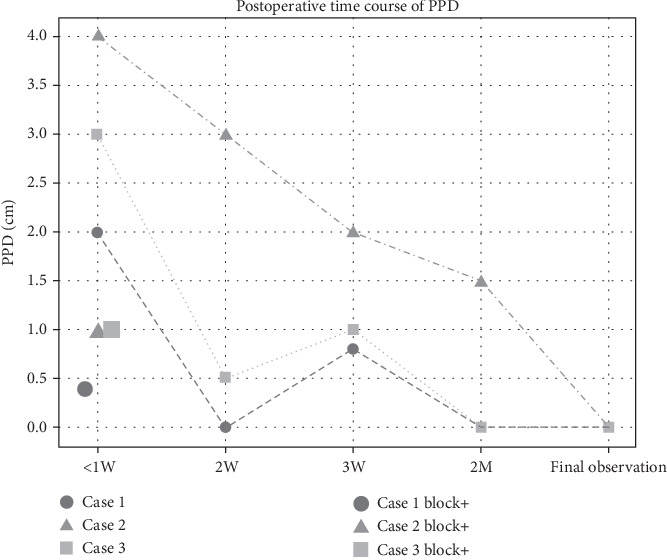
Postoperative changes in pulp–palmar distance (PPD) over time.

Figure 3 shows the postoperative course of PPD. All patients achieved a PPD of 0 cm at the final observation.

The PPD within 1 week improved from 2 to 0.4 cm in Case 1, 4 to 1 cm in Case 2, and 3 to 1 cm in Case 3 before and after PNB. In Cases 1 and 3, the PPD at Week 2 was better than within 1 week after PNB. The PPD at Week 3 increased slightly; however, at 2 months, it was 0 cm. Finally, all patients scored “good” on Flynn's functional evaluation [7]. No adverse events occurred in any of the patients.

## 3. Discussion

We observed early improvements in ROM in patients who underwent PNB. The PPD was within 1 cm in all patients, particularly in the first postoperative week. This also resulted in a good PPD even without PNB in Week 2. However, the worsening PPD at Week 3 compared to that at Week 2 could be attributed to the patient's discharge from the hospital, as they did not receive adequate rehabilitation. Wong et al. [8] observed biological changes in tendon adhesions over time, albeit in a mouse experiment, and reported that the adhesion area was maximal on Day 21. This may also be related to the worsening of the PPD at Week 3.

A systematic review of the management of PFT [9] highlighted the importance of early antimicrobial and surgical interventions. Prevention of tendon adhesions is also important in hand surgery; therefore, early postoperative rehabilitation is necessary [10]. However, if extensive debridement is required and the skin incision is large, postoperative pain may limit active postoperative motion. PNB, which allows hand motion with pain relief, may be effective.

In the original PNB method, a catheter for epidural anesthesia was used for continuous infusion of anesthesia [1, 2]. The difference between the original method and our method is that anesthesia was not continuously injected, and patient-controlled anesthesia was not possible. We used levobupivacaine, a long-acting local anesthetic, instead of a continuous injection to obtain a long effect time. Watanabe et al. employed continuous ulnar NB for active exercise and reported limited MP joint extension due to the blocked interosseous muscles [3]. By contrast, our method did not involve continuous injection, allowing MP joint extension once the anesthetic effect wore off. The original method requires additional procedures, such as a skin incision for catheter placement, whereas our method requires only a single injection or placement of an intravenous catheter under ultrasound guidance. Although our method was less effective than the original method in terms of sustained pain relief, it may be easier to implement.

Our proposed method has some limitations. The wound at the puncture site widened with increased implantation time, and the anesthetic agent could not be delivered subcutaneously. While single-dose administration can solve this problem, it may impose a significant psychological burden on the patient due to the need for frequent needle insertion. The burden is even greater when targeting the ring finger because the median and ulnar nerves must be blocked. The patient's background, affected finger, and healthcare environment should also be considered when selecting the appropriate method of administration. Additionally, all patients had limited PIP joint extensions. The purpose of this block was to relieve pain during flexion. Hence, pain during extension may not be completely relieved, and blocked intrinsic muscles may have hindered the ability to apply force during PIP joint extension.

In conclusion, we reported three cases of postoperative rehabilitation that employed PNB after pyogenic flexor tendinitis. In all cases, good flexion ROM was achieved in the early postoperative period, and the final results were favorable. However, additional measures must be implemented to improve these extensions.

## Figures and Tables

**Table 1:** Patients' data.

**Case**	**Sex**	**Age**	**Cause**	**Affected finger**	**Michon's classification**	**At last observation** **(months)**
1	F	16	Animal bite	Index finger	2	4.5
2	F	81	Sharp trauma	Middle finger	2	30
3	M	49	Sharp trauma	Little finger	2	3

## Data Availability

The data that support the findings of this study are available from the corresponding author upon reasonable request.
